# Psychometric Analysis of a School Social Climate Scale: Input Elements for the Investigation and Promotion of Well-Being

**DOI:** 10.3389/fpsyg.2020.605326

**Published:** 2020-12-18

**Authors:** Mónica Bravo-Sanzana, Edgardo Miranda-Zapata, Horacio Miranda

**Affiliations:** ^1^Núcleo Científico Tecnológico en Ciencias Sociales y Humanidades, Universidad de La Frontera, Temuco, Chile; ^2^Departamento de Producción Agropecuaria, Universidad de La Frontera, Temuco, Chile

**Keywords:** multidimensional, ecological systems theory, structural equation models, psychometric properties, evaluation, wellbeing, school social climate

## Abstract

School social climate from a multidimensional perspective is a focus of great interest in international research and educational and well-being public policies due to the high prevalence of interpersonal violence in adolescents, currently considered a global public health problem. The object of the present study was to assess the psychometric measurement capacity of a set of items to evaluate school social climate in the Student Context Questionnaire of the Chilean Education Quality Measurement System. The sample analyzed consisted of second-year high school students who replied to a Student Context Questionnaire in 2015 (n~158,572). Exploratory and confirmatory factorial analyses were carried out in a measurement model to identify the presence of constructs and items of high homogeneity. The results showed an acceptable to a good fit in the final model, which consisted of 15 latent constructs, and also showed invariance for school administrative dependencies and invariance for sex. All the above implies a contribution to the public organisms that create standardized tests along with the investigation in compulsory secondary education measurement to prevent future violent behaviors, contribute to reducing interpersonal violence, and improve the well-being of the educational community.

## Introduction

School social climate (SSC) has been extensively studied by empirical and theoretical means, especially considering reports of negative factors associated with interpersonal violence in adolescents, which has generated concern in health and education policies (Senanayake et al., 2019). From an ecological perspective, it is understood as the affective and cognitive perceptions of the social interactions, relationships, values, and beliefs of students, teachers, administrators, and personnel in a school (Bronfenbrenner, 1989; Rudasill et al., 2018).

School violence is defined as any physical or psychological assault, or threat of assault, between participants in a school (Akiba et al., 2010). This behavior is present from the earliest years of schooling (Albaladejo-Blázquez et al., 2013) and serves as a predictor of the quality of social coexistence in schools (Córdoba et al., 2016) and academic performance (Bravo-Sanzana et al., 2020). Recent studies have shown that the relation between school attachment and violent behavior is mediated over time by violent attitudes (Varela et al., 2018). Another recent study indicated that adverse childhood experiences increase the risk of violence perpetration and victimization, suggesting that schools should evaluate adverse childhood experiences systematically to increase access to intervention services (Forster et al., 2017).

Furthermore, interpersonal violence in adolescents is sufficiently prevalent to be considered a world public health problem (Senanayake et al., 2019). This category includes other types of violence, such as adolescent dating violence (Vivolo-Kantor et al., 2016) and the school violence suffered by adolescents who are dissatisfied with their socially assigned gender and are at higher risk of staying away from school due to their concerns about safety and their experiences of harassment. This situation requires further research into structural factors such as SSC to orient the development of prevention efforts (Klemmer et al., 2019).

The literature identifies other negative factors associated with school life, such as bullying (Machado et al., 2015; Baldry et al., 2017; Thornberg et al., 2017), cyberbullying (Estévez et al., 2018; López-Castedo et al., 2018), and discrimination (Molla, 2016; Yupanqui et al., 2016; Trucco and Inostroza, 2017).

The creation of this construct has received attention as a way of improving academic performance (Cocorada et al., 2017; Bravo-Sanzana et al., 2019b; Laurito et al., 2019), reducing problem behaviors (Cornell and Huang, 2016; Gaias et al., 2017; Konold et al., 2017; Moratto et al., 2017; Valdés-Cuervo et al., 2018), developing environments with a lower perception of stress and greater effectiveness and job satisfaction among teachers (Malinen and Savolainen, 2016; Bravo-Sanzana et al., 2019a), and especially recognizing the impact of school climate on well-being experience and school engagement (Lombardi et al., 2019). Likewise, school climate is important for promoting student life satisfaction and for preventing the negative consequences associated with being bullied (Lázaro-Visa et al., 2019), as well as the association between SSC and school mental health. Recent reviews and studies report mental health-related factors such as low self-esteem, low motivation, and low interest in going to school, insecurity, and psychiatric problems such as depression, anxiety, and even suicide (Klomek et al., 2010; Suldo et al., 2012; Kutsyuruba et al., 2015; Bravo-Sanzana et al., 2016; Aldridge and McChesney, 2018). In this context, a study on violence against teachers suggests that SSC can play a role in reducing the probability of teacher victimization (Huang et al., 2017). This represents a clear need for proper measurement of this construct to obtain the input information needed by governments and schools to make evidence-based decisions on general and contextual issues.

### School Social Climate From the Perspective of Ecological Systems Theory

Conceiving school space as a type of environment situates us in the complex relations and interrelations of all the social agents and factors involved. In psychological terms, the ecological environment is the factor that most influences human behavior (Bronfenbrenner, 1979). This is a set of nested structures organized at different interacting levels: microsystem, mesosystem, exosystem, and macrosystem, with each level containing the next (Bronfenbrenner, 1987). This ecological model of society has been adapted to the school context by different authors because of its usefulness in understanding social processes within the school (Thapa et al., 2013; Benbenishty et al., 2016).

The concept of SSC is still under construction because no consensus has yet been reached on its definition and the constructs it contains. Its theoretical construct has been little discussed. For example, the review by Thapa et al. (2013) focused on five essential constructs of SSC: safety, relations, teaching and learning, institutional environment, and the school improvement process. However, the recent literature suggests other constructs that have provided empirical evidence about SSC, such as the classroom climate created by teachers (Alonso-Tapia and Nieto, 2019), the effectiveness of teachers in managing student behavior (Malinen and Savolainen, 2016), or components of students' subjective well-being (Benavente et al., 2017). In this context, Rudasill et al. (2018) proposed a theoretical model to orient studies on SSC called Systems View of School Climate, based on the theoretical framework of the Ecological Systems Theory of Bronfenbrenner (1989, 1992). Rudasill et al. (2018) also considered the deconstruction of earlier models and empirical research about SSC to summarize the existing literature, orient research, and provide a widely applicable framework for research into this construct.

This integral framework places individuals, i.e., students, teachers, education assistants, and the like, in the center of a series of nested, interactive contexts (nested structures according to Bronfenbrenner, 1987), which function synergistically to support or discredit students' experiences in school. The authors incorporate nanosystems, a new component for examining interactions between subsystems within schools, such as classrooms.

In this theoretical framework, the microsystem represents the school, the space in which the SSC develops out of the affective and cognitive perceptions of its members and where influential factors converge. Here, nanosystems, e.g., peer groups or sports teams in the school, are nested in the microsystems and are exclusive to the school and each context. The mesosystem is created by the interaction of the school and family microsystems, as well as other factors that influence the SSC. Exosystems, macrosystems, and synchronization systems also include broader factors that can help identify influences in the school climate (Rudasill et al., 2018).

Thus, the SSC construct exists within the school microsystem; however, its formation is complex as it may be the result of multiple influences on the proximal, i.e., personal interactions by direct contact and distal levels of the system where the SSC does not exist, yet these may be considered potential influences on its development. Furthermore, the characteristics of the school's students, teachers, and personnel may be considered factors related to the internal development of the SSC. Other elements form part of the microsystem, such as leadership, teaching practices, and the physical environment, which may be related to perceptions of the SSC. Students' families, the community, other institutions, education policies, and social norms all form part of the theoretical framework as significant variables and additional mechanisms influencing the SSC. Finally, the chronosystem or time (Bronfenbrenner and Ceci, 1994) is incorporated to explain and consider how events in life can direct interactions and influence relations; this could be shown by longitudinal studies of the construct (Rudasill et al., 2018).

### Measuring the Construct

There is a wide range of scales for measuring the SSC; although they present good psychometric evidence, they may lack a broad theoretical basis to support the construct (Trianes et al., 2006; Gálvez Nieto et al., 2014); they may not be based on a multidimensional perspective (Benbenishty and Astor, 2005) or even those with a multidimensional perspective may fail to incorporate an important dimension, such as teacher–student relations (Wang and Degol, 2015) or the student's sense of identity with the school (Elipe et al., 2018). Although there are recent advances in measurement with a solid theoretical base (Gálvez-Nieto et al., 2020), according to Kearney et al. (2020), studies regarding school climate assessment have been marked, for example, by limited sample sizes or narrow developmental levels.

Thus, the SSC construct is complex and must be measured from a multidimensional perspective; this is why social interactions are established at the school level, as schools develop unique environments and at the same time influence social relations and interactions as well as individual behaviors. It is in these unique environments where relevant factors, either positive or negative, emerge to better understand the dynamics of the SSC by providing insight on how to intervene in schools to foster school environments that will promote the learning and the well-being of the educational community.

Based on the theoretical platform presented earlier, this study's object is to make an empirical evaluation of a measurement model to identify the constructs and items of greatest convergence. This has implications for educational policy management and for the empirical and theoretical inputs for investigating SSC.

## Methods

This research is instrumental (Montero and León, 2002), making it a secondary study, quantitative, exploratory, and correlational, with a non-experimental, cross-sectional design (Toro and Parra, 2010).

### Database

The analyzed information was obtained from the database of the Chilean Education Quality Measurement System (SIMCE). The questionnaires were answered by 195,509 second-year high school students (second year of compulsory secondary education, ESO). Of these respondents, 37% presented complete data responding to all the items on all the variables associated with SSC in the Student Context Questionnaires attached to the SIMCE tests. Non-parametric multiple imputations were applied with random forest bootstrap (Stekhoven and Bühlmann, 2012), using the R MissForest package in cases where fewer than 20% of the variables were missing and where no whole item groups for an entire construct were left blank. Thus, the final database for statistical analysis consisted of 158,572 students.

The mean age was 15.33 (SD = 0.602) with a minimum of 14 and a maximum of 19 years. Fifty-one percent were reported as girls and 49% as boys. Of the students, 32.7% were enrolled in schools run by a municipality, whereas 58.8% studied in state-subsidized private schools and 8.5% in fee-paying private schools.

### Instruments

Secondary information was used from data recorded in the Student Context Questionnaires from the SIMCE 2015 (CCES2015); these answers were in pencil and paper format. The questionnaire consisted of 414 items, most of them in ordinal scale responses grouped into 42 categories (Table is attached as complementary material). It was designed to obtain information on the students' school and family environment. It contains indicators in the personal and social spheres, including the SSC, described by the Chilean Education Ministry as school social coexistence, as a management instrument (Agencia de Calidad de la Educación, 2015).

The scales used for this study are related to variables important for evaluating the SSC (see Table 1). Thus, the authors of this study configured scales, categorizing items according to their explicit or implicit origin and compared them with the literature review referring to SSC. Seventy-seven items were selected and grouped into 14 categories, all directly related to SSC factors.

**Table 1:** Description of the SSC constructs according to the SIMCE 2015 evaluation scales.

**School social climate factors reported by the student**	**N^**°**^ in student questionnaire**	**Description**	**Number of items**
Promotion of participation in class	14	Perception of the teacher's promotion of participation in class (expressing opinions, debates, listening respectfully, etc.)	4
Climate in the classroom	15	Perception of respect, order, and cleanliness in the classroom.	6
Climate of trust in the school	17	Perception of trust between people in the school.	5
Discrimination	18	Perception of discrimination in multiple forms: sex, ethnic group, beliefs, etc.	13
School violence	22	Perception of violence in the school in various forms: theft, threats, aggression, etc.	8
Student–teacher violence	23	Perception of student–teacher violence: pushing, insults, mockery, etc.	4
Student safety	24	Perception of how safe the student feels in different spaces in the school: hallways, restrooms, classroom, etc.	5
Bullying	25	Perception of the frequency of intimidation or ill-treatment suffered by the student.	4
Disciplinary measures	26	Perception of the dissemination, intervention, and application of the school's disciplinary rules.	3
Illicit actions in school	30	Perception of the frequency of the consumption of alcohol, tobacco, and drugs in the school.	4
Participation in school activities	36	Perception of the student's participation in school activities.	6
Leadership in school activities	37	Perception of the student's leadership in school activities.	5
Satisfaction with the school	40	Perception of the student's satisfaction with the school.	6
Identity with the school	42	Perception of the student's identity with the school.	4

### Data Analysis

To investigate the existence of evidence supporting the validity of the scale structure, the information was separated into two samples for estimation and validation with 67 and 33%, respectively, as recommended by Xu and Goodacre (2018) using the random simple cross-validation method that corresponds to the most commonly used data splitting method to estimate the exploratory factor analysis (EFA) and validate the confirmatory factorial analysis (CFA). In the first sample, the EFA of the ordinal variables was applied to the polychoric correlation matrix, using unweighted least squares to estimate factors and parallel analysis to determine the number of factors to be retained using the Factor program (Lorenzo-Seva and Ferrando, 2006). In the second sample, the CFA was carried out using the Psych R package (Revelle, 2018), with an unweighted least squares estimation on the polychoric correlation matrix.

To determine the reliability and the internal consistency of the scale, the Cronbach's alpha and McDonald's omega 1999 coefficients and the average variance extracted were calculated.

The following indices were used to assess the fit of the models to the data: comparative fit index (CFI), Tucker–Lewis index (TLI), and root mean square error of approximation (RMSEA). For CFI and TLI, the fit of the model was considered adequate with values higher than 0.90 or 0.95 (Schreiber et al., 2006), whereas for RMSEA, the fit was considered reasonable with values below 0.08 (Hooper et al., 2008).

Finally, to evaluate the scale's stability between male and female students, a measurement invariance analysis was applied to ordinal variables using multigroup analysis. The configural invariance and the measurement invariance, i.e., weak, strong, and strict, were scored according to Dimitrov's 2010 proposals. To determine the satisfaction of the configural invariance, the same goodness-of-fit indices were used as described for CFA. The satisfaction of the different levels of measurement invariance was established when the CFI's delta, i.e., the difference between the CFI of the most and least restricted models, was below 0.01 (Cheung and Rensvold, 2002). The analyses were performed using the Mplus software 7.1 (Muthén and Muthén, 1998–2012).

## Results

The parallel analysis determined the presence of 15 latent constructs based on the selection of the items that did not present crossed loads with respect to a quartimin rotation. Ten items were eliminated because they presented crossed correlations in the exploratory factorial analysis stage. Construct 22, school violence, was separated into two subdimensions: direct school violence such as fights, threats, or physical aggression and indirect school violence such as theft, ill-intentioned rumors, mockery, or insults. As a result of the EFA, the scale was established with 15 constructs and 67 items. When this structure was fitted to the data in a CFA in the estimation sample, good goodness-of-fit indices were obtained (RMSEA = 0.021; CFI = 0.984; TLI = 0.982), reflecting the good fit of the model to the data. The estimations of the standardized factorial loads of the items were statistically significant and presented values higher than 0.40. The results are presented in Table 2 and the reliability statistics and omegas in Table 3.

**Table 2:** Constructs of SSC and saturations of the items of which it is composed. All loadings are significant (*P* ≤ 0.001).

**N^**°**^**	**Construct**	**N^**°**^ Item**	**Description**	**Loading**
1	Participation in the classroom		During this year, 2015, how often have the following situations occurred in your class?	
		1	The teachers have promoted student participation in the classroom.	0.67
		2	The teachers have stimulated students to express their opinions.	0.76
		3	The teachers have encouraged students to listen to and respect the opinions of their classmates.	0.72
		4	Debates have been organized in class on issues of public interest.	0.49
2	Climate in the classroom		How frequently have the following situations occurred this year, 2015?	
		1	The students have respected one another.	0.74
		3	The students in my class have respected the teachers.	0.70
		6	The students in my class have made sure the classroom is clean.	0.48
3	Climate of trust in the school		How frequently have the following situations occurred this year, 2015?	
		2	I have felt sufficient trust to approach my teachers.	0.71
		3	I have felt sufficient trust to approach a school director or authority (e.g., inspector, adviser, director, etc.).	0.63
		4	I have felt that my school is a welcoming, friendly place.	0.89
4	Discrimination		During this year, 2015, how frequently have you felt that people in school look down on you, discriminate against you, or exclude you for one of the following reasons?	
		1	Your sex (because you are a boy or a girl).	0.66
		4	Your sexual orientation.	0.62
		6	Your religion.	0.60
		7	Your political ideas.	0.66
		9	You suffer a disability.	0.59
		10	Your family's economic situation.	0.66
		11	The ethnic group or culture to which you belong.	0.64
		12	You are an immigrant, or your parents are immigrants.	0.52
		13	You are pregnant or have children.	0.50
5	Direct school violence		How frequently have the following situations occurred this year, 2015?	
		3	Fights between students (e.g., shouting, screaming, pushing, hair-pulling, punching, etc.).	0.74
		5	Threats or harassment between students.	0.78
		6	Threats or aggression with knives or pen-knives, knuckle-dusters, nunchucks, etc.	0.63
		7	Threats or aggression with firearms.	0.47
		8	Students breaking or damaging the school (e.g., breaking benches, windows, chairs, computers, etc.).	0.69
6	Indirect school violence		During this year, 2015, how often have the following situations occurred in your school?	
		1	Theft inside the school.	0.64
		2	Ill-intentioned rumors, isolation (“sending someone to Coventry”) between students.	0.63
		4	Insults, bad language, mockery, and dismissive behavior between students.	0.72
7	Student–teacher violence		During this year, 2015, how often have the following situations occurred between teachers and students in your school?	
		1	Students pushing or hitting a teacher.	0.54
		2	Teachers pushing or hitting a student.	0.49
		3	Insults, bad language, mockery, and dismissive behavior of teachers by students.	0.75
		4	Insults, bad language, mockery, and dismissive behavior of students by teachers.	0.61
8	Student safety		During this year, 2015, how safe or unsafe have you felt in the following parts of your school?	
		1	School entrances and exits.	0.80
		2	Classroom.	0.87
		3	Hallways.	0.91
		4	Yards.	0.90
		5	Restrooms.	0.88
9	Bullying		During this year, 2015, how frequently have other students at your school intimidated you or maltreated you in the following ways?	
		1	Physically.	0.68
		2	Verbally.	0.80
		3	Socially.	0.76
		4	Electronically.	0.67
10	Disciplinary measures		During this year, 2015, how frequently have your teachers, the inspector, or the director carried out the following actions?	
		1	Intervened in situations of maltreatment and intimidation between students.	0.82
		2	Applied the procedures of the school coexistence manual in situations of maltreatment and intimidation between students (e.g., interview with parent or guardian, punishments, etc.).	0.94
		3	Has it been explained to all the students what they should do in situations of maltreatment or intimidation?	0.68
11	Illicit actions in school		During this year, 2015, how frequently has a student at your school carried out the following actions?	
		1	Smoke cigarettes during school hours (e.g., smoking in the restrooms during breaks).	0.79
		2	Drink alcohol during school hours (e.g., beer, wine, pisco, etc.).	0.74
		3	Consume drugs during school hours (e.g., cannabis, based paste, amphetamines, etc.).	0.84
		4	Offer drugs to other students in the school (e.g., cannabis, based paste, amphetamines, etc.).	0.83
12	Participation in school activities		During this year, 2015, how often have you participated in the following activities in your school?	
		1	I have participated in activities marking the start and end of academic periods	0.70
		2	I have participated in commemorative activities (e.g., Independence Day celebrations, religious ceremonies, etc.).	0.73
		3	I have participated in recreational activities (e.g., bingos, festivities, competitions, etc.).	0.74
		5	I have participated in academic and cultural activities (e.g., plays, art exhibitions, science, and technology fairs, debating competitions, etc.).	0.66
		6	I have participated in volunteer or community service campaigns (e.g., raising money or food, planting trees, cleaning up the school, etc.).	0.70
13	Leadership in school activities		During this year, 2015, how often have you helped to organize or carry out the following activities in your school?	
		1	I have helped to organize commemorative activities.	0.85
		2	I have helped to organize recreational activities.	0.87
		3	I have helped to organize sports activities.	0.70
		4	I have helped to organize academic and cultural activities.	0.81
		5	I have helped to organize volunteer or community service campaigns.	0.81
14	Satisfaction with the school		How satisfied or dissatisfied are you with your school in each of the following aspects?	
		1	The quality of the education given in the school.	0.77
		2	The academic preparation given in the school.	0.76
		3	The values taught in the school.	0.78
		4	The school infrastructure (e.g., classrooms, restrooms, yards, etc.).	0.62
		5	The relation between classmates in the school.	0.75
		6	The relation between teachers and students in the school.	0.77
15	Identity with the school		How strongly do you agree with each of the following statements about your school?	
		1	I feel proud of my school.	0.90
		2	I speak well of my school to other people.	0.86
		3	If someone spoke ill of my school, I would defend it.	0.76
		4	I would recommend changing to my school to a friend.	0.77

**Table 3:** Reliability and alpha and omega coefficients.

**Dimension**	**Alpha**	**Omega**	**AVE**
Promotion of participation in class	0.74	0.75	0.43
Climate in the classroom	0.66	0.66	0.40
Climate of trust in the school	0.80	0.78	0.55
Discrimination	0.85	0.84	0.39
Direct school violence	0.80	0.82	0.50
Indirect school violence	0.69	0.70	0.45
Student–teacher violence	0.70	0.72	0.44
Student safety	0.94	0.94	0.76
Bullying	0.81	0.83	0.57
Disciplinary measures	0.85	0.86	0.67
Illicit actions in school	0.87	0.88	0.65
Participation in school activities	0.83	0.83	0.50
Leadership in school activities	0.90	0.90	0.65
Satisfaction with the school	0.88	0.88	0.54
Identity with the school	0.89	0.89	0.67

*AVE, average variance extracted*.

Each scale presented good levels of reliability, with Cronbach's alpha values between 0.66 and 0.94 and McDonald's omega values between 0.66 and 0.94. The average variance extracted presented values below 0.50 in 5 out of the 12 constructs; the lowest value was 0.39, which implies that more than half of the variance of these constructs is not explained by the items of which it is composed (Table 3).

The measurement invariance analysis by students' sex showed that strict invariance was satisfied (Table 4). This establishes the stability of the scale measurement in students of both sexes, meaning that the results obtained in each dimension can be compared between groups by sex. Any variation between groups in any item is due only to variations in the latent variable of each group.

**Table 4:** Measurement invariance of items about SSC by sex in estimation and validation samples.

		**Estimation sample**					**Validation sample**	
	**Configural**	**Metric**	**Scalar**	**Strict**	**Configural**	**Metric**	**Scalar**	**Strict**
CFI	0.947	0.944	0.939	0.932	0.945	0.942	0.937	0.930
CFI delta	NA	0.003	0.005	0.007	NA	0.003	0.004	0.006

The CFA with the validation sample showed that the model presented a good fit with the data (RMSEA = 0.015; CFI = 0.990; TLI = 0.989) and good levels of reliability, with omega and alpha values between 0.66 and 0.93 for the constructs of the scale. The measurement invariance analysis by students' sex also showed that strict invariance was satisfied (Table 4). This corroborated the stability of the scale measurement in students of both sexes, allowing the results obtained in each dimension to be compared between groups by sex.

The model presents a good fit for every school administrative dependency category. The CFA for municipal, i.e., public, schools shows good fit indices (RMSEA = 0.027; CFI = 0.910; TLI = 0.905). The same holds for state-subsidized private schools (RMSEA = 0.025; CFI = 0.914; TLI = 0.909). In the case of fee-paying private schools, the fit is poorer (RMSEA = 0.025; CFI = 0.891; TLI = 0.885) but acceptable; there may be a sample size effect here, as this type of school administration is smaller than the other school administration sample sizes. In fact, the sample size of municipal schools is 21,827, state-subsidized private schools 40,260, and fee-paying private schools 5,461. Nonetheless, the measurement invariance analysis shows regular fit indices for configural invariance, with a good RMSEA level but poor CFI and TLI (RMSEA = 0.044; CFI = 0.859; TLI = 0.853). Nevertheless, the CFI deltas show a slight decrease of fit across the measurement invariance levels (Table 5), reaching the strict measurement invariance. This implies the model can be used through the different school administrative dependencies.

**Table 5:** Measurement invariance of items about SSC by school administrative dependency in the validation sample.

	**Configural**	**Metric**	**Scalar**	**Strict**
CFI	0.856	0.852	0.847	0.843
CFI delta	NA	0.004	0.005	0.004

## Discussion

The object of this study was to assess the psychometric measurement capacity of a set of items to evaluate the SSC in the SIMCE through exploratory and confirmatory factorial analyses to identify the presence of constructs and items with high homogeneity or convergence.

For the constructs Promotion of Participation, Climate in the Classroom, Discrimination, Student-Teacher Violence, and Indirect School Violence, more than half of the variance could not be explained by the items of which they are composed, indicating that they need to be reviewed by the managers of the SIMCE standardized test. Special mention must be made of the construct School Violence, one of the most important variables for academic performance in Latin American countries, particularly Chile (LLECE, 2008; Trucco and Inostroza, 2017) and one of the most frequently reported variables in the study of the construct. This variable was divided into two constructs alluding to an explicitly aggressive form of violence: Direct School Violence; and School Violence expressed in rumors, threats, and theft: Indirect School Violence. These constructs operate on the SSC scale and contribute together with the rest, presenting a 56% variance, which gives them good fit and reliability. The present study provides a good basis for future work with this and the other constructs.

Based on this study's results, it may be concluded that the instrument measures SSC adequately, showing the existence of differentiation in the constructs, which theoretically configure this construct with different degrees of correlation.

The model with 15 correlated factors indicates that the SSC comprises different processes, all closely related, but which can be reported separately (Lara et al., 2018).

The instrument presents good internal consistency in each of the constructs indicated, allowing students at risk of SSC to be identified. Results offer input for the orientation of school improvement projects and others related to managing social coexistence in school. From this perspective, significantly low evaluations on the scale may indicate adverse school environments (Moratto et al., 2017), and it will be possible to identify which of the constructs require intervention in school planning. In turn, the significantly high scores allow the school to identify factors that could be considered strengths and areas that are still important for orienting management of the school's social coexistence policy.

Tables 6, 7 show quartiles for the total scale and subscales, respectively. They were calculated on the sum of the answers to the items that comprise each subscale and the sum of all subscales (DiStefano et al., 2009).

**Table 6:** Quartiles for the total scale.

**Scale**	**Range**	**CSE level**	**Category/description**
SSC	0-Q1	Very bad	Negative climate, with alert in critical general areas
	Q1-Q2	Bad	Negative climate, with alert in many critical areas to improve.
	Q2-Q3	Good	Positive climate, with some areas to improve.
	Q3-Max.	Very good	Positive climate, with emphasis in sustainability management.

**Table 7:** Quartiles for subscales.

	**Percentiles**		
	25	50	75
Promotion of participation in class	10	11	13
Climate in the classroom	7	8	9
Climate of trust in the school	7	9	10
Discrimination	9	9	10
Direct school violence	6	8	10
Indirect school violence	6	7	9
Student–teacher violence	4	4	6
Student safety	15	15	19
Bullying	4	4	5
Disciplinary measures	7	9	12
Illicit actions in school	4	5	8
Participation in school activities	8	11	14
Leadership in school activities	5	8	11
Satisfaction with the school	17	18	21
Identity with the school	10	12	14

The SSC measurement provides both educational centers and local government with inputs that will enable them to establish a school's network strategies to work cooperatively on social coexistence and assess the effectiveness of intervention programs (MINEDUC, 2015).

One of the strengths of this study is that the system was validated with a sample of Chilean adolescents belonging to schools of the three types existing in the Chilean educational system, i.e., public, subsidized private, and fee-paying private, and considering the type of teaching; therefore, it is representative, and the results are reliable. In addition, results also show invariance for school administrative dependencies and invariance for sex. This implies the model can be used through the different types of schools and with equal accuracy in both sexes.

A second strength is that the instrument represents a wide range of factors related to the SSC, giving it a more comprehensive view of the construct.

One of the study's limitations is the need not only to measure the SSC from the student's perception but also to incorporate other key actors of the educational community to identify contextual factors.

## Conclusion

The results illustrated the adjustment and reliability values of the measure and factorial invariance across school administrative dependencies and invariance for sex.

We conclude that the SSC scale introduced in the SIMCE 2015 Student Context Questionnaire permits greater understanding of the SSC construct and enables public policy and research into measuring SSC to make decisions based on reliable and valid input to generate other instruments, programs, and interventions that will help to ensure improvements and well-being in school environments.

Future work should focus on exploring the perceptions of school climate of other key actors in the educational community to identify contextual factors and, in addition, linking findings from the scale to other key school variables such as social–emotional competency.

## Data Availability Statement

The data analyzed in this study is subject to the following licenses/restrictions: The databases of the Chilean Education Quality System, SIMCE, are confidential, they cannot be shared. Requests to access these datasets should be directed to monicaviviana.bravo@ufrontera.cl.

## Ethics Statement

The studies involving human participants were reviewed and approved by 170/15 Comité de Ética de la Universidad de La Frontera. Written informed consent from the participants' legal guardian/next of kin was not required to participate in this study in accordance with the national legislation and the institutional requirements.

## Author Contributions

MB-S created the research question, conducted a bibliographic search, theoretical framework, methodological design, and contributed to the discussion. EM-Z contributed methodological design, performed the data analysis, and generated the results. HM contributed to the methodological design and data analysis. All authors contributed to the article and approved the submitted version.

## Conflict of Interest

The authors declare that the research was conducted in the absence of any commercial or financial relationships that could be construed as a potential conflict of interest.
